# Changes in the incidence of pneumonia, bacterial meningitis, and infant mortality 5 years following introduction of the 13-valent pneumococcal conjugate vaccine in a "3+0" schedule

**DOI:** 10.1371/journal.pone.0183348

**Published:** 2017-08-16

**Authors:** Sylvia Becker-Dreps, Bryan Blette, Rafaela Briceño, Jorge Alemán, Michael G. Hudgens, Gilberto Moreno, Ana Ordoñez, Julio Rocha, David J. Weber, Erick Amaya

**Affiliations:** 1 Department of Family Medicine, University of North Carolina School of Medicine, Chapel Hill, North Carolina, United States of America; 2 Department of Biostatistics, UNC Gillings School of Global Public Health, Chapel Hill, North Carolina, United States of America; 3 Sistemas Locales de Atención Integral a la Salud, León (SILAIS-León), León, Nicaragua; 4 Hospital Escuela Oscar Danilo Rosales Argüello (HEODRA), León, Nicaragua; 5 Division of Infectious Diseases, University of North Carolina School of Medicine, Chapel Hill, North Carolina, United States of America; 6 Department of Microbiology and Parasitology, Faculty of Medical Sciences, National Autonomous University of Nicaragua, León, Nicaragua; Public Health England, UNITED KINGDOM

## Abstract

**Background:**

*Streptococcus pneumoniae* causes about 826,000 deaths of children in the world each year and many health facility visits. To reduce the burden of pneumococcal disease, many nations have added pneumococcal conjugate vaccines to their national immunization schedules. Nicaragua was the first country eligible for GAVI Alliance funding to introduce the 13-valent pneumococcal conjugate vaccine (PCV13) in 2010, provided to infants at 2, 4, and 6 months of age. The goal of this study was to evaluate the population impact of the first five years of the program.

**Methods:**

Numbers of visits for pneumonia, pneumonia-related deaths, and bacterial meningitis in both children and adults, and infant deaths between 2008 and 2015 were collected from all 107 public health facilities in León Department. Vital statistics data provided additional counts of pneumonia-related deaths that occurred outside health facilities. Adjusted incidence rates and incidence rate ratios (IRRa) in the vaccine (2011–2015) and pre-vaccine periods (2008–2010) were estimated retrospectively using official population estimates as exposure time.

**Results:**

The IRRa for pneumonia hospitalizations was 0.70 (95% confidence interval [CI]: 0.66, 0.75) for infants, and 0.92 (95% CI: 0.85, 0.99) for one year-olds. The IRRa for post-neonatal infant mortality was 0.56 (95% CI: 0.41, 0.77). In the population as a whole, ambulatory visits and hospitalizations for pneumonia, as well as pneumonia-related mortality and rates of bacterial meningitis were lower in the vaccine period.

**Conclusions:**

During the first five years of program implementation, reductions were observed in health facility visits for pneumonia in immunized age groups and infant mortality, which would be hard to achieve with any other single public health intervention. Future study is warranted to understand whether the lack of a booster dose (e.g., at 12 months) may be responsible for the small reductions in pneumonia hospitalizations observed in one year-olds as compared to infants.

## Introduction

In 2015, pneumonia was among the leading causes of child mortality in the world, responsible for an estimated 921,000 deaths of children under age five [1]. In children and adults, *S*. *pneumoniae* is the most commonly identified infectious cause of community-acquired pneumonia [2, 3]. To reduce the burden of pneumococcal disease, many high-income countries have rolled out immunization programs with pneumococcal conjugate vaccines (PCV). In December 2010, Nicaragua, became the first nation eligible for funding by the GAVI Alliance to add the 13-valent pneumococcal vaccine (PCV13) to its national immunization schedule. The vaccine was introduced using a “3+0” schedule with three primary doses provided to infants at 2, 4, and 6 months of age, but no booster dose at 12–15 months of age. By the end of 2015, 53 other GAVI-eligible nations have also introduced PCV [4]; the majority of these nations also follow a 3+0 schedule.

Evidence to support the effectiveness of a 3+0 schedule includes a clinical trial with the nine-valent pneumococcal conjugate vaccine (PCV9) in the Gambia. In that trial, PCV9 was 37% efficacious against radiological pneumonia, and 3 to 4 years after the primary series, children receiving PCV9 had higher pneumococcal antibody titers against 3 of the 9 serotypes included in the vaccine as compared to children in the placebo group [5, 6]. Also, Australia introduced PCV7 using a 3+0 schedule in 2005 [7] and during the first 30 months of the program, observed a 38% reduction in all-cause pneumonia hospitalization rates among children under age two years. However, an immunogenicity study comparing the 3+0, 2+1, and 3+1 schedules showed that infants randomized to receive the 3+0 schedule had lower IgG titers against all pneumococcal polysaccharides at 13 months of age as compared to the 2+1 or 3+1 schedules [8]. Further, in a case-control study, a 3+1 schedule was found to be slightly more protective against invasive pneumococcal disease than a 3+0 schedule [9].

The goal of this study was to evaluate the continued impact of a PCV13 immunization program provided in a 3+0 schedule in a Gavi-eligible nation. During the first two years (2011 and 2012) after PCV13 introduction in Nicaragua, reductions were observed in the rates of health facility visits for pneumonia among immunized age groups; among older adults, lower pneumonia-related mortality was observed, likely due in part to herd protective effects [10, 11]. Now that the program has been in place for five years, this new study re-examines these outcomes in all age groups over additional years, as well as changes in the rates of uncommon outcomes, such as pneumonia-related mortality, bacterial meningitis, and infant mortality.

## Methods

### Setting

Data were collected between 2008 and 2015 in the Department of León, Nicaragua (official 2015 population: 410,860). Nicaragua is a lower middle income country with a per-capita gross domestic product of US$ 2,087 in 2015 [12]. The tropical climate of Nicaragua includes rainy and dry seasons; an annual peak of pneumonia cases typically occurs during the rainy season months of July to November. As in many low and middle income countries, there is little ongoing laboratory surveillance for pneumococcal disease.

Beginning on December 12, 2010, PCV13 was offered to infants at two, four, and six months of age as part of the national immunization program. During the first year of the program, a single catch-up dose of vaccine was offered to children between six and 24 months of age. PCV13 is administered as part of well child visits at all health centers and health posts. Hib vaccines were added to the pediatric immunization schedule in 1999. During all years studied, influenza vaccines were provided to children under the age of 36 months with chronic illnesses, pregnant women, and adults over the age of 50 years with chronic illnesses; in 2010, the H1N1 vaccine was offered separately from the seasonal influenza vaccine. After 2010, H1N1 was offered combined with the seasonal influenza vaccine. In addition, beginning in early 2010, adults aged 50 years and older with chronic illnesses were offered the 23-valent pneumococcal vaccine (PPSV23); in 2011, individuals aged 2 to 49 years with chronic illnesses also became eligible for PPSV23. Coverage of PPSV23 and influenza vaccines in adults > 50 years of age between 2008 and 2012 was previously reported [11]; in 2013, 2014, and 2015, respectively, 24.3%, 20.9%, and 25.6% of adults aged > 50 years received PPSV23, and 5.6%, 12.4%, and 9.9% of adults aged > 50 years received influenza vaccines. Influenza and PPSV23 vaccines are offered through yearly vaccination campaigns.

### Data collection

Counts of health facility visits for pneumonia, pneumonia-related deaths, bacterial meningitis, and infant deaths were collected by the Ministry of Health in León from public health facilities in all 10 municipalities in the Department. The 107 health facilities included a 400-bed referral hospital, 13 *centros de salud* (large primary care centers with several physicians on staff), and 93 *puestos de salud* (small primary care posts often with a single general physician on staff). Hospital admissions trended upward slightly during the years examined, with a mean of 15,095 yearly admissions prior to PCV13 introduction and a mean of 16,531 yearly admissions following PCV13 introduction.

At the hospital and *centros de salud*, physicians provided counts of visits with these diagnoses to the on-site health statistician, while at the *puestos de salud*, the physicians provided counts directly to the epidemiologist at the Ministry of Health in León. Visits were categorized as hospitalizations or ambulatory care visits; ambulatory care visits included visits to primary care centers and health posts, and visits to the emergency department which did not result in hospital admission. Outside of public health facilities, the Ministry of Health estimates that 10% of the population receives care in private health facilities.

Separately, counts of deaths attributable to pneumonia were collected from the Office of Vital Statistics in the Department of León. Any pneumonia-related deaths that were reported to the Office of Vital Statistics, but were not captured by the health facilities were added to the total numbers of pneumonia-related deaths. This study of deidentified data was approved by the Institutional Review Board of the University of North Carolina at Chapel Hill.

### Assignment of diagnosis codes

In the hospital setting, diagnoses were assigned at the time of admission by the hospital physician in charge of the admission. For patients treated in the emergency department without hospitalization, diagnoses were assigned by the emergency department physician treating the patient. Physicians were trained to use the definition of pneumonia defined in the Ministry of Health’s Guide to the Management of Common Infectious Diseases in Childhood and Malnutrition (p. 81) [13] for children, and in the Management Protocol of Common Medical Problems in Adults (p. 72) [14], as an infectious syndrome including constitutional and respiratory symptoms, present with physical exam findings of consolidation, with confirmation of infiltrate on chest radiograph. Clinical guidelines for both children and adults require that patients hospitalized for pneumonia receive a chest radiograph. Physicians were trained to use the following definition of bacterial meningitis (p.170) [13]: physical exam findings of nuchal rigidity (positive Kernig or Brudzinski signs), petechiae or purpura, change in mental status (including lethargy or irritability), or signs of increased intracranial pressure (such as bulging fontanelle in infants) together with greater than 1,000 leukocytes/μL of cerebrospinal fluid with a predominance of polymorphonuclear leukocytes. While physicians were trained to use the above definitions, the final assignment of diagnosis is based on the treating physician’s clinical judgement.

If a patient dies of pneumonia in any health facility, a report of a pneumonia-related death is recorded. For any death occurring outside of any health facility, a team including a Ministry of Health physician, the state epidemiologist, and the local medical provider investigate and assign the cause of death.

We also examined infant deaths without a reportable cause to potentially capture deaths due to undiagnosed pneumococcal infections in this low resource setting. We did not include infant deaths with a reportable cause (including pneumonia, diarrhea, dengue, tuberculosis, leptospirosis, HIV, child abuse, homicide, motor vehicle collision, poisoning) in this sum to avoid double counting infant deaths (in the case of pneumonia), and to exclude causes of death which clearly did not result from pneumococcal infection.

As a comparison diagnosis that should not be affected by PCV13 introduction, we examined changes in health facility visits for diarrhea, which physicians were trained to define as an increase in stool frequency and volume to three or more stools within a 24 hour period (p.127) [13]. The rotavirus vaccine was first introduced in 2006, and coverage was high and remained constant during the years studied (2008–2015). In addition to the diagnosis code, each report includes the patient’s gender, age group, the week and year of the visit, and the patient’s municipality of origin.

In the primary care centers and health posts, visit diagnoses are assigned by the physician treating the patient. Pneumonia is diagnosed using the same definitions as in the hospital setting, with the exception that chest radiography is rarely accessible in the community, and therefore, the diagnosis relies primarily on physical exam findings. Counts are provided to the Department’s epidemiology office, where they are maintained in an electronic database. As in the hospital setting, each report includes the patient’s gender, age group, the week and year of the visit, and the patient’s municipality of origin.

### Population estimates

The Ministry of Health provides official population estimates for each municipality in León by age group and year. The total official population in 2015 for the Department of León was 410,860. These estimates are based on the national census, conducted by the Nicaraguan Institute of Development Information; the last census was conducted in 2005. Population estimates for subsequent years are projected using data from birth certificates, school enrollment, and death certificates [15].

### PCV13 coverage

The Departmental Office of the National Immunization Program maintains data on immunizations administered by age group and year for PCV13. By the end of 2011, 63% of infants had received three doses of PCV13 (range by municipality: 49% to 71%) and 87% of one year-olds had received a catch-up dose of PCV13 (range by municipality: 68% to 100%). In 2012, 97% of infants had received three doses of PCV13 (range by municipality: 80% to 100%) [10] and since 2012, high coverage has been maintained, with a range of 89% to 100% by municipality.

### Statistical analyses

Annual incidence rates of pneumonia hospitalizations, ambulatory visits for pneumonia, pneumonia-related mortality, bacterial meningitis, and infant mortality were estimated from 2008 to 2015. Our primary focus was on infants and one year-olds (children aged 12 to 23 months), as these age groups in Nicaragua have a high burden of pneumococcal disease, and were also eligible to receive PCV13 during the years studied.

We first extracted numbers of health facility visits for pneumonia between January 2005 to December 2015 and calculated the numbers of visits by week to examine trends in pneumonia visits during the past decade. Due to a change in health care policy that reduced the cost of health care visits beginning in 2007, for further analysis, the time period was limited to 2008–2015. Numbers of health facility visits for pneumonia, pneumonia-related deaths, bacterial meningitis, and infant deaths were examined. Counts of hospital admissions for pneumonia were retrieved from the hospital epidemiology database. Emergency department visits that did not result in hospital admission were categorized as ambulatory visits, in addition to visits to primary care centers or health posts.

Incidence rates of pneumonia hospitalizations, ambulatory visits for pneumonia, pneumonia-related mortality, bacterial meningitis, infant mortality, and corresponding 95% confidence intervals (CI) in the vaccine and pre-vaccine periods were estimated using generalized estimating equations (GEE), to allow for correlation of individuals living within a municipality. Exposure time was estimated by official municipality population estimates for each calendar year and age group.

Incidence rates were compared in the years after vaccine introduction to the years before introduction. For individuals under age 50 years, we defined “pre-vaccine”, as 2008–2010, and “vaccine” as 2011–2015, as PCV13 was introduced in December 2010, and therefore, the vaccine’s impact could first be measured in 2011. For older age groups, we dropped 2010 from the analysis as a transitional year, since PPSV23 was offered to individuals with chronic diseases in early 2010, and pediatric PCV13 was introduced in late 2010. As a comparison diagnosis, we examined incidence rates of all health facility visits for diarrhea over the same years studied above. Incidence rate ratios (IRR) and 95% CI were also estimated using GEE for the vaccine period as compared to the pre-vaccine period. In these analyses, we controlled for municipality as categorized into one of three groups: 1) urban (León municipality), 2) peri-urban (municipalities that border León municipality), and rural (municipalities that do not border León municipality), to account for distance from a patient's home municipality to the hospital (located in León municipality) and potential differences in care seeking and immunization coverage by municipality category.

## Results

### Health facility visits for pneumonia

**Fig 1:**
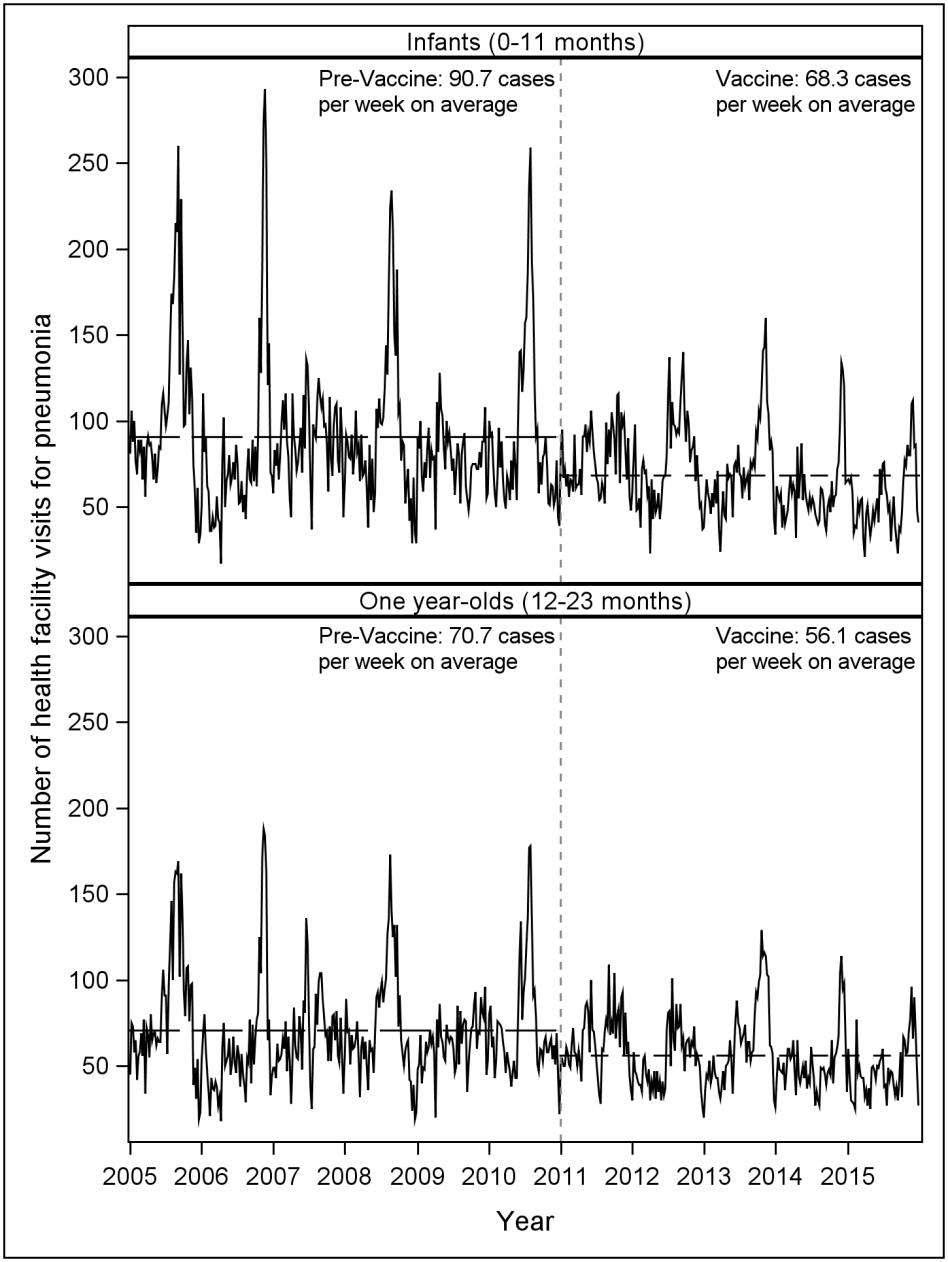
Total numbers of health facility visits for pneumonia in infants and one year-olds by week, 2005–2015. Dashed vertical lines represent date of initiation of PCV13 immunization program.

Between 2005 and 2015, the total numbers of health facility visits for pneumonia by week for infants and one year-olds are shown in Fig 1.

### Ambulatory visits for pneumonia

**Fig 2:**
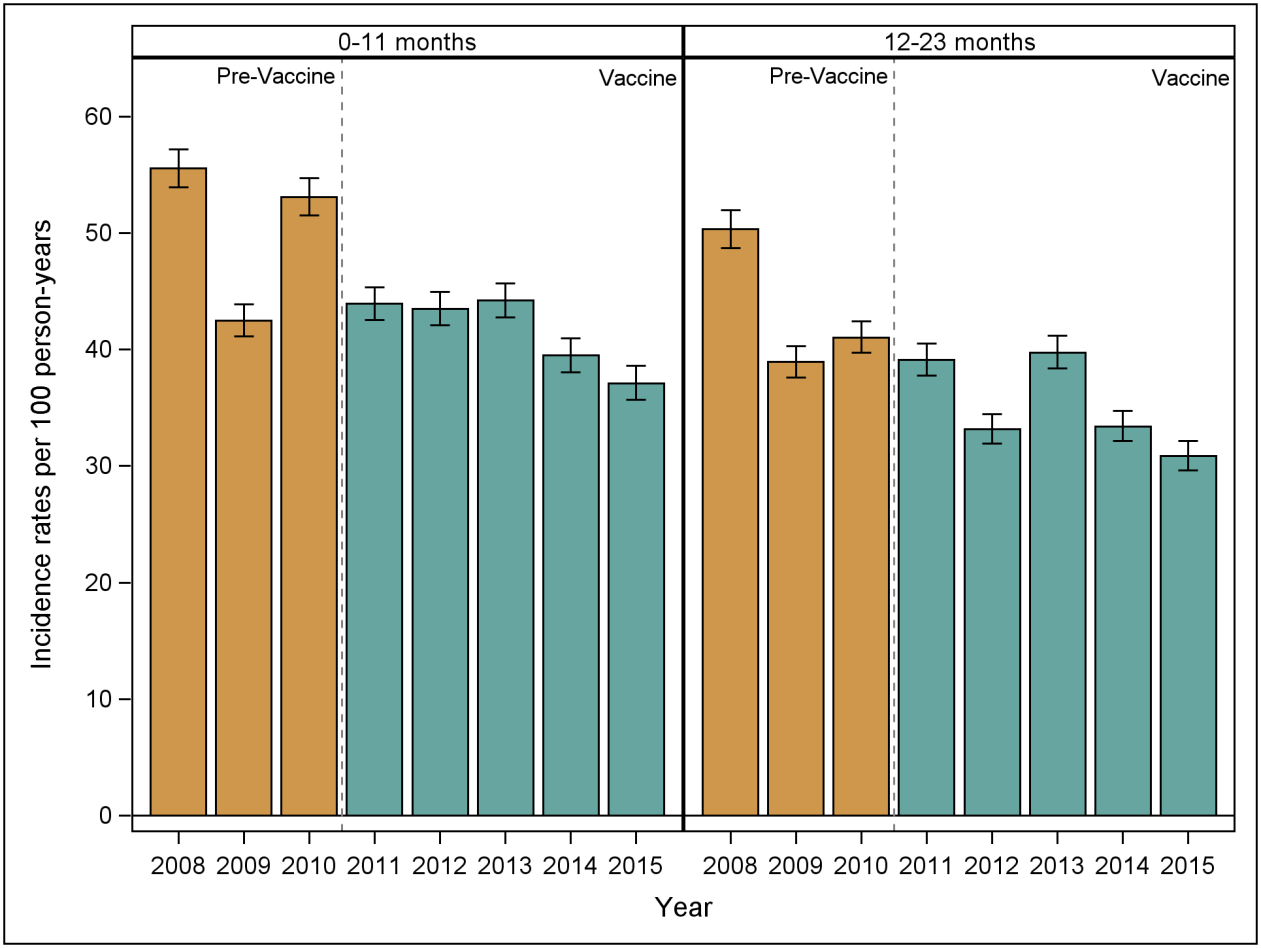
Incidence rates of ambulatory visits for pneumonia in infants and one year-olds by year, 2008–2015.

The numbers of ambulatory visits for pneumonia in the pre-vaccine and vaccine periods by age group are shown in Table 1. Incidence rates of ambulatory visits for pneumonia by year for infants and one year-olds are shown in Fig 2. The estimated IRRa for ambulatory visits for pneumonia in the vaccine period as compared to the pre-vaccine period was 0.84 (95% CI: 0.74, 0.96) among infants, and 0.82 (95% CI: 0.70, 0.96) among one year-olds. The estimated IRRa for ambulatory visits for other age groups are shown in Table 2. Note that the total IRR estimate is less than each age group specific IRR estimate; in general this type of result can occur because the rate ratio is not collapsible [16].

**Table 1:** Numbers and incidence rates of health facility visits for pneumonia, pneumonia-related deaths, and bacterial meningitis cases in the pre-vaccine and vaccine periods in León, Nicaragua.

	<12 months^a^		12 to 23 months^a^		24 to 59 months^a^		5 to 14 years^a^		15 to 49 years^a^		50 to 64 years^b^		≥ 65 years^b^		Total	
	Pre-vaccine	Vaccine	Pre-vaccine	Vaccine	Pre-vaccine	Vaccine	Pre-vaccine	Vaccine	Pre-vaccine	Vaccine	Pre-vaccine	Vaccine	Pre-vaccine	Vaccine	Pre-vaccine	Vaccine
Ambulatory visits for pneumonia (cases per 1,000 person-years)	12,278 (501.3)	16,071 (418.7)	10,443 (431.7)	13,746 (353.6)	11,702 (165.9)	16,458 (140.3)	5,753 (23.2)	8,280 (21.7)	3,555 (5.6)	5,415 (4.9)	637 (8.9)	1,697 (7.8)	554 (13.0)	1,495 (12.3)	44,922 (40.1)	63,162 (31.3)
Hospitalizations for pneumonia (cases per 1,000 person-years)	1,575 (64.3)	1,743 (45.4)	600 (24.8)	888 (22.8)	509 (7.2)	806 (6.9)	191 (0.8)	310 (0.8)	175 (0.3)	259 (0.2)	32 (0.4)	118 (0.5)	90 (2.1)	307 (2.5)	3,172 (2.8)	4,431 (2.2)
Pneumonia-related deaths (cases per 100,000 person-years)	23 (93.9)	37 (96.4)	1 (4.1)	3 (7.7)	4 (5.7)	6 (5.1)	3 (1.2)	1 (0.3)	38 (5.9)	33 (3.0)	14 (19.6)	31 (14.2)	63 (148.1)	103 (84.5)	146 (13.0)	214 (10.6)
Bacterial meningitis hospitalizations^c^	2	3	0	0	2	1	4	0	14	5	2	1	1	1	25	11
Exposure time in person-years	24,491	38,382	24,193	38,878	70,556	117,327	247,617	380,981	639,445	1,103,835	71,432	217,617	42,529	121,946	1,120,263	2,018,966

^a^Pre-vaccine: January 2008 to December 2010; Vaccine: January 2011 to December 2015.

^b^Pre-vaccine: January 2008 to December 2009; Vaccine January 2011 to December 2015

^c^Incidence not displayed for bacterial meningitis due to low number of cases per age group. Overall incidence was 2.2 cases per 100,000 person-years in the Pre-vaccine period and 0.5 cases per 100,000 person-years in the Vaccine period.

**Table 2:** Adjusted incidence rate ratios (IRRa) of health facility visits for pneumonia and diarrhea (comparison diagnosis) in the vaccine period vs. the pre-vaccine period^a^.

Age group	Ambulatory visits for pneumonia	Hospitalizations for pneumonia	Diarrhea visits^b^ (comparison)
0–11 months	0.84 (0.74, 0.96)^c^	0.70 (0.66, 0.75)	1.25 (0.93, 1.67)
12–23 months	0.82 (0.70, 0.96)	0.92 (0.85, 0.99)	1.34 (0.98, 1.83)
24–59 months	0.85 (0.71, 1.01)	0.95 (0.82, 1.10)	1.69 (1.14, 2.50)
5–14 years	0.94 (0.82, 1.07)	1.05 (0.97, 1.14)	2.08 (1.39, 3.10)
15–49 years	0.88 (0.73, 1.07)	0.85 (0.76, 0.96)	2.48 (1.53, 4.02)
50–64 years	0.88 (0.68, 1.12)	1.21 (0.91, 1.61)	1.79 (1.22, 2.64)
65+ years	0.94 (0.74, 1.21)	1.19 (1.15, 1.23)	1.31 (1.10, 1.57)
Total	0.78 (0.68, 0.90)	0.77 (0.73, 0.82)	1.56 (1.04, 2.34)

^a^Pre-vaccine years: 2008–2010; Vaccine years: 2011–2015. For analyses in adults ≥50 years, 2010 is excluded as a transitional year.

^b^Includes both ambulatory visits and hospitalizations for diarrhea.

^c^Corresponding 95% confidence intervals

### Pneumonia hospitalizations

**Fig 3:**
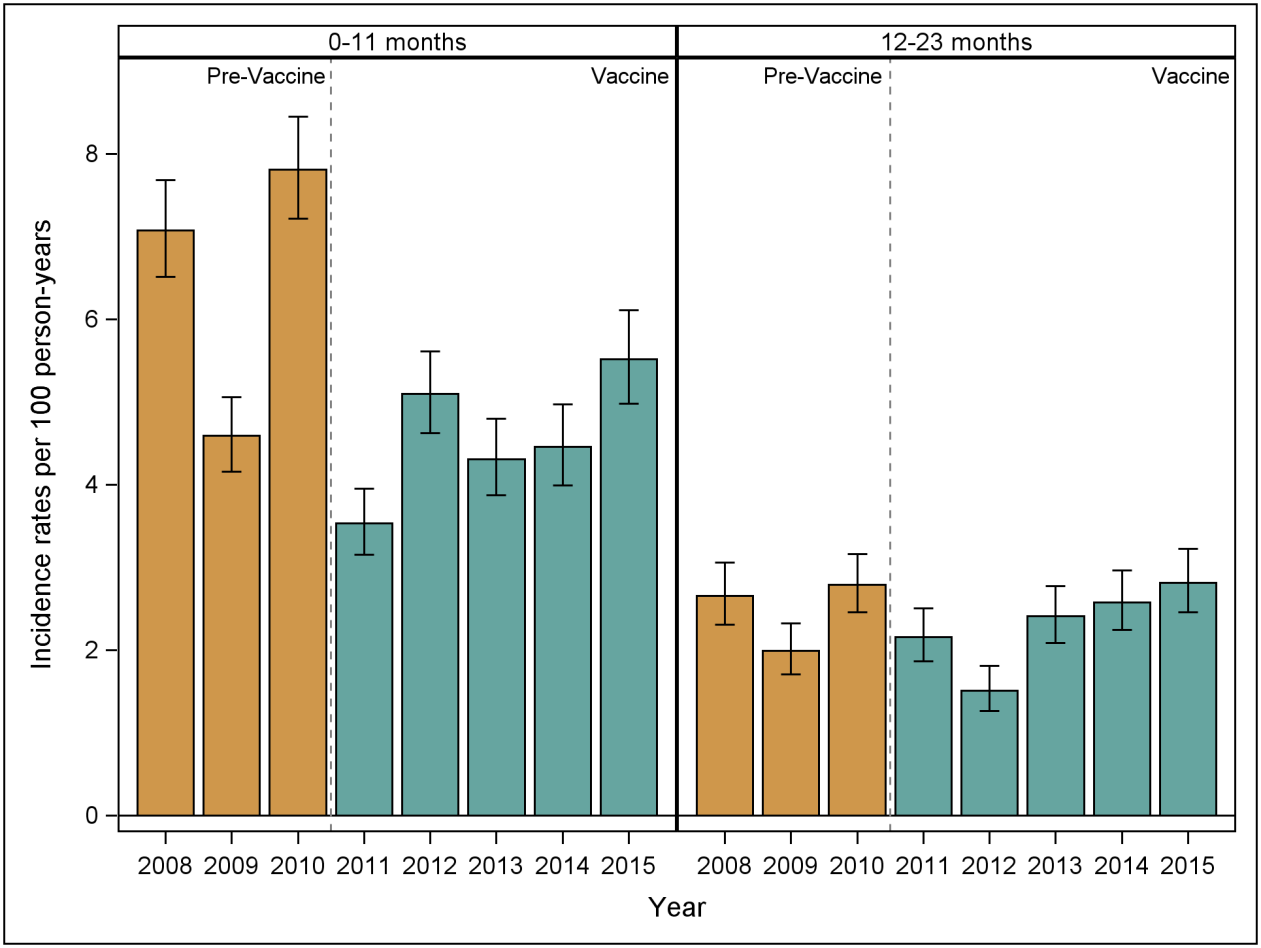
Incidence rates of pneumonia hospitalizations in infants and one year-olds by year, 2008–2015.

The numbers of pneumonia hospitalizations in the pre-vaccine and vaccine years are shown by age group in Table 1. Incidence rates of pneumonia hospitalizations by year for infants and one year-olds are shown in Fig 3. The estimated IRRa for pneumonia hospitalizations was 0.70 (95% CI: 0.66, 0.75) among infants, and 0.92 (95% CI: 0.85, 0.99) among one year-olds. IRRa for other age groups are shown in Table 2.

### Health facility visits for diarrhea

As a comparison diagnosis, we include IRRa estimates for health facility visits for diarrhea in the same years examined above (Table 1). The estimated IRRa for health facility visits for diarrhea was 1.25 (0.93, 1.67) among infants, and 1.34 (0.98, 1.83) among one year-olds. In the population as a whole, there was a significant increase in the incidence rate of diarrhea visits in the vaccine period as compared to the pre-vaccine period (Table 2).

### Pneumonia-related mortality

The numbers of deaths attributed to pneumonia by age group are shown in Table 1. Changes in pneumonia-related mortality in the vaccine vs. the pre-vaccine period (IRRa) are shown in Table 3. In this table, age groups were categorized as young child (0 to 4 years), child (5 to 14 years), adult (15 to 49 years), and older adult (≥ 50 years), due to low numbers of events. Overall pneumonia-related mortality in the population was lower in the vaccine period as compared to the pre-vaccine period (IRRa = 0.81 [95% CI: 0.76, 0.86]), but did not change significantly for children. As compared to the pre-vaccine period, pneumonia-related mortality was lower in the vaccine period for adults aged 15 to 49 years (IRRa = 0.50 [95% CI 0.39, 0.64]) and adults aged 50 years and older (IRRa = 0.58 [95% CI, 0.53, 0.64]).

**Table 3:** Adjusted incidence rate ratios (IRRa) of pneumonia-related mortality and bacterial meningitis in the vaccine period vs. the pre-vaccine period^a^.

Age Group	Pneumonia-related mortality	Bacterial Meningitis
0–4 years	1.01 (0.67, 1.52)^b^	0.62 (0.15, 2.47)
5–14 years	0.22 (0.02, 2.47)	0 (0, 0.98)^c^
15–49 years	0.50 (0.39, 0.64)	0.21 (0.07, 0.57)
≥ 50 years	0.58 (0.53, 0.64)	0.22 (0.04, 1.34)
Total	0.81 (0.76, 0.86)	0.24 (0.12, 0.50)

^a^Pre-vaccine years: 2008–2010; Vaccine years: 2011–2015. For analysis in adults ≥50 years, 2010 is excluded as a transitional year.

^b^Corresponding 95% confidence intervals

^c^Due to no counts in the vaccine period, unadjusted results are presented in this age group.

### Bacterial meningitis

The numbers of hospitalizations for bacterial meningitis by age group are shown in Table 1. The IRRa of bacterial meningitis by age group are shown in Table 3. Overall, bacterial meningitis was a rare occurrence. There was a significant reduction in bacterial meningitis in the vaccine period in the population as a whole (IRRa = 0.24 [95% CI: 0.12, 0.50], and among those aged 5 to 49 years.

### Infant mortality

**Fig 4:**
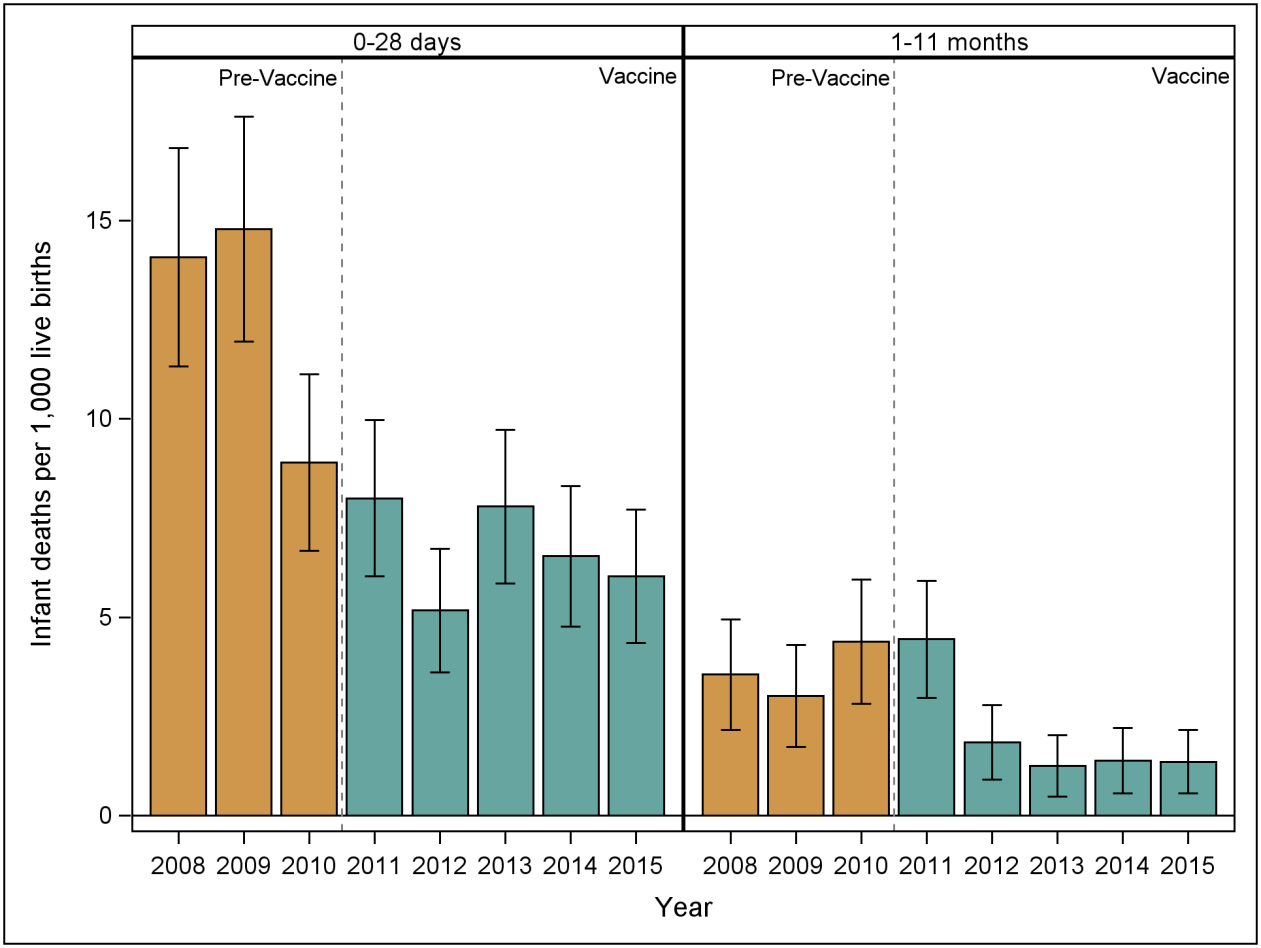
Incidence rates of infant deaths without a reportable cause among neonates and infants ages 1 to 11 months, 2008–2015.

In the pre-vaccine period there were 339 total infant deaths without a reportable cause in León among 20,850 live births recorded between 2008 and 2010. Of these infant deaths, 263 were among neonates and 76 were among infants aged 1 to 11 months. In the vaccine period there were 422 infant deaths among 40,037 live births recorded between 2011 and 2015. Of these infant deaths, 301 were among neonates and 121 were among infants aged 1 to 11 months. The incidence rate of infant deaths without a reportable cause between 2008 and 2015 is shown for neonates and infants aged 1 to 11 months in Fig 4. The IRRa for infant deaths among neonates in the vaccine vs. pre-vaccine period was 0.53 (95% CI: 0.45, 0.63) and among infants aged 1 to 11 months was 0.56 (95% CI: 0.41, 0.77).

## Discussion

During the first five years of a pediatric PCV13 immunization program using a 3+0 schedule which attained high coverage, there were reductions in hospitalizations and ambulatory visits for all-cause pneumonia among immunized age groups. Infants experienced a 30% reduction in the incidence of pneumonia hospitalizations in the vaccine period as compared to the pre-vaccine years. This reduction was similar to that reported among children under two years of age in Australia after introduction of PCV7 also in a 3+0 schedule [7], and in the US after introduction of a “3+1” schedule, with doses of vaccine provided at 2, 4, 6, and 12–15 months of age [17]. However, among one year-olds in our study, the reduction in the incidence of pneumonia hospitalizations was not as substantial as compared to infants, estimated at 8%. In comparison, a study of all-cause pneumonia hospitalizations in Uruguay following introduction of a PCV7/PCV13 immunization program in a “2+1” schedule (doses provided at 2, 4, and 12 months) found similar reductions in all-cause pneumonia hospitalizations among infants and one year-olds (52% and 54%, respectively) [18]. In Nicaragua, it is possible that the lack of a booster dose after the primary series may be responsible for decreased duration of protection in the 12 to 23 month old age group. This hypothesis is supported by evidence of decreased anti-polysaccharide IgG titers in the second year of life among children who received PCV in a 3+0 schedule, as compared to a 2+1 or 3+1 schedule [8]. Further investigation is warranted to understand whether the small reduction in pneumonia hospitalizations observed in the 12 to 23 month age group could be attributed to the 3+0 schedule. If the 2+1 schedule is found to provide better protection to one year-olds, the greater effectiveness of the schedule must be weighed against potential differences in vaccine coverage for the third dose between the two schedules.

While we did not observe a reduction in pneumonia-related mortality among young children, it is probable in this setting that some pneumonia-related deaths among infants were instead categorized as infant deaths. Infant mortality was substantially lower in the vaccine period, as compared to before PCV13 introduction. Upon examination of the distribution of infant deaths, similar reductions were observed in neonates as in infants aged 1 to 11 months. While pneumococcal disease does occur in neonates [19, 20], any change in neonatal mortality due to PCV13 would be due to indirect rather than direct effects. It would be more likely that PCV13 would have an effect on infant deaths in the post-neonatal age group.

There were substantial reductions in pneumonia-related mortality among adults. For example, adults aged 50 years and older experienced a 42% lower incidence of pneumonia-related deaths in the vaccine period as compared to the pre-vaccine period. This reduction may be in due in part to indirect effects from the pediatric PCV13 immunization program, as was observed among older age groups in the US following PCV7 introduction [21]. In addition, the simultaneous introduction of a PPSV23 immunization program for those with chronic illnesses could also be contributing to these reductions, although coverage of PPSV23 among the overall population of older adults was low [11] and there is not clear prior evidence that PPSV23 reduces mortality in adults [22].

Hospitalization for bacterial meningitis was rare in the population, possibly due to low access to laboratory diagnostic testing. Despite overall low rates of bacterial meningitis, all IRRa estimates show a decreased incidence of bacterial meningitis in the vaccine period as compared to the pre-vaccine period, with statistically significant decreases observed for those aged 5 to 49 years. A decrease in the incidence of bacterial meningitis has been observed in both high and low income countries following PCV introduction [23, 24].

Limitations of this study include that there was not laboratory surveillance in this setting to determine whether cases of pneumonia and bacterial meningitis were due to *S*. *pneumoniae* or other pathogens. However, since *S*. *pneumoniae* is a common cause of these infections in both adults and children [25, 26], a substantial reduction of the portion due to *S*. *pneumoniae* is likely to be detected in the all-cause incidence of these infectious syndromes. Laboratory surveillance conducted in other Latin American countries showed that PCV13 would cover between 77% and 88% of the serotypes causing invasive pneumococcal disease in the region [27]. Finally, as with any observational study, we cannot prove that reductions observed were due to the vaccine, and not to other factors that differed in the two time periods, for example, changes in access to health facilities over time. To investigate this further, we estimated incidence rates of health facility visits for diarrhea, which would not be affected by the introduction of PCV13. We found that diarrhea visits actually increased in the vaccine years, which may indicate an increased incidence of diarrhea, or that access to health facilities improved over time. If the latter is true, then our estimates of changes in health facility visits for pneumonia may be conservative.

In summary, five years following introduction of a pediatric PCV13 program in a 3+0 schedule, there were substantial reductions observed in health facility visits for pneumonia in immunized age groups and infant mortality, which would be hard to achieve with any other single public health intervention. In the population as a whole, reductions were observed in pneumonia-related mortality and rates of bacterial meningitis following PCV13 introduction. Future study is warranted to better understand the effectiveness of the 3+0 schedule in the 12 to 23 month age group, who carry a high burden of pneumococcal disease.

## References

[1] Liu L, Oza S, Hogan D, Chu Y, Perin J, Zhu J, et al Global, regional, and national causes of under-5 mortality in 2000–15: an updated systematic analysis with implications for the Sustainable Development Goals. Lancet. 2016; 388(10063): 3027–3035. doi: 10.1016/S0140-6736(16)31593-8 27839855 10.1016/S0140-6736(16)31593-8 PMC5161777

[2] O'Brien KL, Wolfson LJ, Watt JP, Henkle E, Deloria-Knoll M, McCall N, et al Burden of disease caused by *Streptococcus pneumoniae* in children younger than 5 years: global estimates. Lancet. 2009; 374: 893–902. doi: 10.1016/S0140-6736(09)61204-6 19748398 10.1016/S0140-6736(09)61204-6

[3] Said MA, Johnson HL, Nonyane BA, Deloria-Knoll M, O'Brien KL; AGEDD Adult Pneumococcal Burden Study Team, et al Estimating the burden of pneumococcal pneumonia among adults: a systematic review and meta-analysis of diagnostic techniques. PLoS One. 2013; 8(4): e60273 doi: 10.1371/journal.pone.0060273 23565216 10.1371/journal.pone.0060273 PMC3615022

[4] Gavi. The Vaccine Alliance. 2016. In: Gavi Pneumococcal Vaccine Support [Internet]. Geneva; Washington, DC. http://www.gavi.org/support/nvs/pneumococcal/.

[5] Cutts FT, Zaman SM, Enwere G, Jaffar S, Levine OS, Okoko JB, et al Efficacy of nine-valent pneumococcal conjugate vaccine against pneumonia and invasive pneumococcal disease in The Gambia: randomised, double-blind, placebo-controlled trial. Lancet. 2005; 365(9465): 1139–46. 10.1016/S0140-6736(05)71876-6 15794968

[6] Akinsola AK, Ota MO, Enwere GC, Okoko BJ, Zaman SM, Saaka M, et al Pneumococcal antibody concentrations and carriage of pneumococci more than 3 years after infant immunization with a pneumococcal conjugate vaccine. PLoS One. 2012; 7(2): e31050 doi: 10.1371/journal.pone.0031050 22363544 10.1371/journal.pone.0031050 PMC3282700

[7] Jardine A, Menzies RI, McIntyre PB. Reduction in hospitalizations for pneumonia associated with the introduction of a pneumococcal conjugate vaccination schedule without a booster dose in Australia. Pediatr Infect Dis J. 2010; 29(7): 607–12. 20589980 10.1097/inf.0b013e3181d7d09c

[8] Givon-Lavi N, Greenberg D, Dagan R. Immunogenicity of alternative regimens of the conjugated 7-valent pneumococcal vaccine: a randomized controlled trial. Pediatr Infect Dis J. 2010; 29(8): 756–62. doi: 10.1097/INF.0b013e3181d99345 20661103 10.1097/INF.0b013e3181d99345

[9] Whitney CG, Pilishvili T, Farley MM, Schaffner W, Craig AS, Lynfield R, et al Effectiveness of seven-valent pneumococcal conjugate vaccine against invasive pneumococcal disease: a matched case-control study. Lancet. 2006; 368(9546): 1495–502. doi: 10.1016/S0140-6736(06)69637-2 17071283 10.1016/S0140-6736(06)69637-2

[10] Becker-Dreps S, Amaya E, Liu L, Moreno G, Rocha J, Briceño R, et al Changes in childhood pneumonia and infant mortality rates following introduction of the 13-valent pneumococcal conjugate vaccine in Nicaragua. Pediatr Infect Dis J. 2014; 33(6): 637–42. doi: 10.1097/INF.0000000000000269 24445827 10.1097/INF.0000000000000269

[11] Becker-Dreps S, Amaya E, Liu L, Hudgens M, Rocha J, Briceño R, et al Impact of a combined child and adult pneumococcal immunization program on adult pneumonia incidence and mortality in Nicaragua. Vaccine. 2015; 33(1): 222–7. doi: 10.1016/j.vaccine.2014.10.073 25444795 10.1016/j.vaccine.2014.10.073

[12] World Bank. GDP per capita. 2016. In: World Bank Data [Internet]. http://data.worldbank.org/indicator/NY.GDP.PCAP.CD

[13] Nicaraguan Ministry of Health. Guide for the management of the most common infectious diseases in childhood and malnutrition. 11 March, 2011. In: Nicaraguan Ministry of Health [Internet]. http://www.minsa.gob.ni/index.php/repository/Descargas-MINSA/Dirección-General-de-Regulación-Sanitaria/Normas-Protocolos-y-Manuales/Normas-2009/normativa---017-Guía-para-el-Abordaje-de-las-Enfermedades-Infecciosas--más-Comunes-de-la-Infancia-y-la-Destrución/

[14] Nicaraguan Ministry of Health. Guide for the management of the most common medical problems in Adults, September, 2010. 2011. In: Nicaraguan Ministry of Health [Internet]. http://www.minsa.gob.ni/index.php/repository/Descargas-MINSA/Direcci%C3%B3n-General-de-Regulaci%C3%B3n-Sanitaria/Normas-Protocolos-y-Manuales/Normas-2010/Normativa---051-Protocolo-de-atenci%C3%B3n-de-problemas-m%C3%A9dicos-m%C3%A1s-frecuentes-en-adultos/

[15] Instituto Nacional de Información de Desarrollo (INIDE). Metodología de las proyecciones de población con el uso de variables sintomáticas, 2012. In: INIDE, Estadísticas Sociodemográficas. http://www.inide.gob.ni/

[16] Greenland S. Absence of confounding does not correspond to collapsibility of the rate ratio or rate difference. Epidemiology. 1996; 7(5):498–501. 8862980

[17] Grijalva CG, Nuorti JP, Arbogast PG, Martin SW, Edwards KM, Griffin MR. Decline in pneumonia admissions after routine childhood immunisation with pneumococcal conjugate vaccine in the USA: a time-series analysis. Lancet. 2007; 369(9568): 1179–86. doi: 10.1016/S0140-6736(07)60564-9 17416262 10.1016/S0140-6736(07)60564-9

[18] Hortal M, Estevan M, Laurani H, Iraola I, Meny M; Paysandú/Salto Study Group. Hospitalized children with pneumonia in Uruguay: pre and post introduction of 7 and 13-valent pneumococcal conjugated vaccines into the National Immunization Program. Vaccine. 2012; 30(33): 4934–8. doi: 10.1016/j.vaccine.2012.05.054 22664222 10.1016/j.vaccine.2012.05.054

[19] Billings ME, Deloria-Knoll M, O'Brien KL. Global Burden of Neonatal Invasive Pneumococcal Disease: A Systematic Review and Meta-analysis. Pediatr Infect Dis J. 2016; 35(2): 172–9. doi: 10.1097/INF.0000000000000955 26517330 10.1097/INF.0000000000000955

[20] Assandri E, Amorín B, Gesuele JP, Algorta G, Pírez MC. Pneumococcal invasive disease in newborns before and after 7-valent and 13-valent universal pneumococcal vaccination in Uruguay. Rev Chilena Infectol. 2015; 32(2): 167–74. doi: 10.4067/S0716-10182015000300005 26065449 10.4067/S0716-10182015000300005

[21] Griffin MR, Zhu Y, Moore MR, Whitney CG, Grijalva CG. U.S. hospitalizations for pneumonia after a decade of pneumococcal vaccination. N Engl J Med. 2013; 369(2): 155–63. doi: 10.1056/NEJMoa1209165 23841730 10.1056/NEJMoa1209165 PMC4877190

[22] Falkenhorst G, Remschmidt C, Harder T, Hummers-Pradier E, Wichmann O, Bogdan C. Effectiveness of the 23-Valent Pneumococcal Polysaccharide Vaccine (PPV23) against Pneumococcal Disease in the Elderly: Systematic Review and Meta-Analysis. PLoS One. 2017;12(1):e0169368 doi: 10.1371/journal.pone.0169368 28061505 10.1371/journal.pone.0169368 PMC5218810

[23] Bijlsma MW, Brouwer MC, Kasanmoentalib ES, Kloek AT, Lucas MJ, Tanck MW, et al Community-acquired bacterial meningitis in adults in the Netherlands, 2006–14: a prospective cohort study. Lancet Infect Dis. 2016; 16(3): 339–47. doi: 10.1016/S1473-3099(15)00430-2 26652862 10.1016/S1473-3099(15)00430-2

[24] Gatera M, Uwimana J, Manzi E, Ngabo F, Nwaigwe F, Gessner BD, et al Use of administrative records to assess pneumococcal conjugate vaccine impact on pediatric meningitis and pneumonia hospitalizations in Rwanda. Vaccine. 2016; 34(44): 5321–5328. doi: 10.1016/j.vaccine.2016.08.084 27639280 10.1016/j.vaccine.2016.08.084

[25] Marrie TJ, Durant H, Yates L. Community-acquired pneumonia requiring hospitalization: 5-year prospective study. Rev Infect Dis. 1989;11(4):586 2772465 10.1093/clinids/11.4.586

[26] Selwyn BJ. The epidemiology of acute respiratory tract infection in young children: comparison of findings from several developing countries. Coordinated Data Group of BOSTID Researchers. Rev Infect Dis. 1990; 12 Suppl 8: S870–88. 2270410 10.1093/clinids/12.supplement_s870

[27] Castañeda E, Agudelo CI, Regueira M, Corso A, Brandileone MC, Brandão AP, et al Laboratory-based surveillance of Streptococcus pneumoniae invasive disease in children in 10 Latin American countries: a SIREVA II project, 2000–2005. Pediatr Infect Dis J. 2009; 28(9): e265–70. doi: 10.1097/INF.0b013e3181a74b22 19710581 10.1097/INF.0b013e3181a74b22

